# Microtubules are organized independently of the centrosome in *Drosophila *neurons

**DOI:** 10.1186/1749-8104-6-38

**Published:** 2011-12-06

**Authors:** Michelle M Nguyen, Michelle C Stone, Melissa M Rolls

**Affiliations:** 1 Department of Biochemistry and Molecular Biology, The Pennsylvania State University, University Park, PA 16802, USA

## Abstract

**Background:**

The best-studied arrangement of microtubules is that organized by the centrosome, a cloud of microtubule nucleating and anchoring proteins is clustered around centrioles. However, noncentrosomal microtubule arrays are common in many differentiated cells, including neurons. Although microtubules are not anchored at neuronal centrosomes, it remains unclear whether the centrosome plays a role in organizing neuronal microtubules. We use *Drosophila *as a model system to determine whether centrosomal microtubule nucleation is important in mature neurons.

**Results:**

In developing and mature neurons, centrioles were not surrounded by the core nucleation protein γ-tubulin. This suggests that the centrioles do not organize functional centrosomes in *Drosophila *neurons *in vivo*. Consistent with this idea, centriole position was not correlated with a specific region of the cell body in neurons, and growing microtubules did not cluster around the centriole, even after axon severing when the number of growing plus ends is dramatically increased. To determine whether the centrosome was required for microtubule organization in mature neurons, we used two approaches. First, we used *DSas-4 *centriole duplication mutants. In these mutants, centrioles were present in many larval sensory neurons, but they were not fully functional. Despite reduced centriole function, microtubule orientation was normal in axons and dendrites. Second, we used laser ablation to eliminate the centriole, and again found that microtubule polarity in axons and dendrites was normal, even 3 days after treatment.

**Conclusion:**

We conclude that the centrosome is not a major site of microtubule nucleation in *Drosophila *neurons, and is not required for maintenance of neuronal microtubule organization in these cells.

## Background

Centrosomes are the best-studied microtubule-organizing center. At the core of the centrosome are a mother and a daughter centriole, with each centriole composed of nine doublet microtubule sets in *Drosophila*, and variations on this arrangement in other organisms [1]. The centrioles are surrounded by pericentriolar material, which is a dynamic pool of proteins necessary for microtubule anchoring and nucleation [2]. In mitotic animal cells, centrosomes are the focus of the mitotic spindle. In interphase cells, however, the role of the centrosome is more variable. In cultured mammalian cells, centrosomes focus the microtubules into radial arrays; the minus ends remain at the site of nucleation at the centrosome, while the plus ends, at which most subunit addition occurs, grow out to the cell periphery [2,3]. However, in certain differentiated cells, such as neurons and epithelial cells, microtubules are not anchored at the centrosome, although the centrosome may still be present [4,5]. We use *Drosophila *neurons to ask whether the centrosome continues to play a role in organizing microtubules in a differentiated cell with a noncentrosomal microtubule array.

Neurons are an ideal system in which to study the organization of noncentrosomal microtubule arrays because their axons and dendrites contain linear arrays of microtubules. The two compartments are specialized; the axon sends signals to other neurons and other cells of the organism, while the dendrite receives signals. The compartments also have different cytoskeletal organization. Axonal microtubules are arranged with plus ends distal to the cell body (plus-end-out) in all systems that have been studied [6]. In mammalian cultured neurons, dendrites have mixed orientation near the cell body and a uniform plus-end-out arrangement in distal dendrites [6-9]. *Drosophila *neurons *in vivo *have a very simple and highly polarized arrangement, in which dendritic microtubules are oriented with minus ends distal to the soma [10]. In both mammals and *Drosophila*, dendrites are differentiated from axons by the presence of minus-end-out microtubules.

The arrangement of microtubules in neurons is very different from the radial microtubule arrays generated by the centrosome. Nevertheless, the centrosome has been proposed to play a major role in organizing neuronal microtubules. For example, the centrosome has been proposed to serve as the site of microtubule nucleation. After nucleation, microtubules could be severed by katanin and then transported into axons and dendrites in a polarized manner [11]. Several pieces of evidence support this model. Studies on cultured rat sympathetic neurons showed beautifully that the centrosome could nucleate and release microtubules [12]. Moreover, microtubules nucleated at the centrosome could be shown to be transported into the axon [13]. Later, the microtubule-severing protein katanin was found concentrated at neuronal centrosomes and shown to play a role in releasing microtubules from the centrosome [14]. These studies and several others led to a model in which microtubules generated at the neuronal centrosome are transported into axons and dendrites, and this transport of microtubule pieces both provides material for new microtubules and also determines polarity of microtubules in axons and dendrites [6,11,15]. However, most of the studies that led to this model were performed in very young neurons. Other studies in more mature cultured neurons have shown that γ-tubulin, the core of the microtubule nucleation complex, is not concentrated at centrosomes [16,17], calling into question the role of the centrosome in microtubule nucleation in mature neurons. Moreover, ablation of the centrosome did not seem to impair axon growth or regeneration [16]. However, another recent study showed that signaling proteins localized at neuronal centrosomes could control dendrite morphology [18], suggesting that the centrosome may continue to be functionally important in relatively mature neurons. The role of the centrosome in positioning axon outgrowth has been even more controversial, with studies in various cell types showing that it either is important for positioning the nascent axon or plays no role in this process [19]. No *in vivo *studies have examined the impact of the centrosome on microtubule organization or polarity.

In the current study, we test the role of the centrosome in developing and mature neurons *in vivo*, including their role in maintaining microtubule organization. We analyzed *Drosophila *neurons in embryos and larvae to determine whether the centrosome is essential for organizing neuronal microtubules. We found that the core microtubule nucleation protein γ-tubulin was absent from centrioles even at the earliest stages of axon outgrowth, and that the centriole could be located anywhere in the cell body during axon and dendrite outgrowth and in mature neurons. This suggests that the centriole does not organize a centrosome in *Drosophila *neurons at any point in their differentiation. We also examined the trajectories of growing microtubules in neurons and found that, unlike proliferating cells, they do not emanate from the centriole, and that this is the case even when we injure the neuron to cause an upregulation of growing microtubules. In order to further test whether the centriole plays any role in microtubule organization in neurons, we analyzed neurons in *DSas-4 *mutant animals. We used cilia formation in ciliated sensory neurons as a readout of centriole function in these animals. We found that cilia are defective in *DSas-4 *larval sensory neurons, but that non-ciliated larval sensory neurons have normal dendrite structure and microtubule organization. To confirm that the centriole is not required for microtubule organization in mature neurons, we used laser ablation, and found that even 3 days after centriole destruction microtubule organization was normal. We thus conclude that the centrosome does not play an essential role in maintaining neuronal polarity in non-ciliated cells *in vivo*.

## Results

### Centrioles have variable position in developing and mature neurons

Since the centrosome is a major microtubule-organizing center, we wished to test whether it controls microtubule polarity in *Drosophila *neurons, which have plus-end-out microtubules in axons and minus-end-out microtubules in dendrites. We focused on the dendritic arborization (da) sensory neurons, in which microtubules are straightforward to visualize, and in which microtubule polarity studies have previously been performed [10].

**Figure 1:**
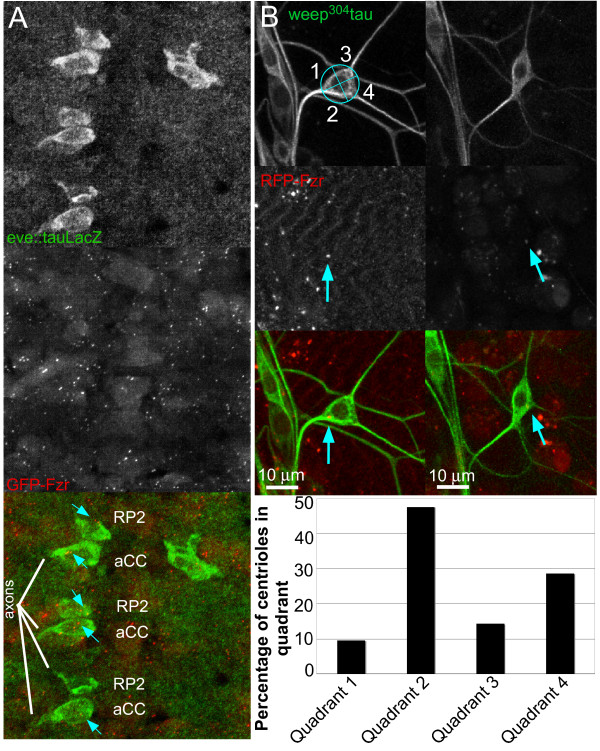
**Centriole localization is not consistent with a role in microtubule organization in *Drosophila *neurons**. **(A) **The centriole does not occupy a stereotypic position in developing aCC and RP2 motor neurons. Stage 12 embryos expressed GFP-Fzr in all cells, and aCC and RP2 were labeled with tau-LacZ reporter expression. GFP puncta within neurons are indicated with arrows. **(B) **Centriole localization in mature neurons is variable. Live imaging of da neurons of larvae expressing red fluorescent protein (RFP)-Fzr (arrows) and tau-GFP was performed. Z-projections are shown. An example of quadrant segregation is shown at the top right. Localization of 21 Fzr puncta within cell bodies was analyzed and quantified.

If centrosomes play a major role in organizing neuronal microtubules in developing or mature neurons, we hypothesized that the centriole might occupy a reproducible position in the neuron. To test whether the centriole has a stereotypic position in neurons, we analyzed the localization of the centriole in embryonic and larval neurons. As the centriole has previously been reported to localize near sites of axon outgrowth in *Drosophila *neurons [20], we wished to examine neurons at the time when the axon started to emerge from the cell body. To visualize neurons early in their development, we used a transgenic *Drosophila *line that expresses a tagged microtubule-binding protein in two motor neurons, aCC and RP2 [21]. We crossed this line with a line that expresses the centriolar marker green fluorescent protein (GFP)-Fzr in all cells [22]. In most *Drosophila *neurons, the axon grows first, and the dendrites extend later. This is true of aCC and RP2, which can be seen to extend axonal processes laterally in stage 12 embryos (Figure 1A). At the time when these nascent axons started to grow, the localization of Fzr varied within the cell body relative to the new axon (Figure 1A).

We performed a similar experiment with mature neurons after completion of axon and dendrite growth. Using the red fluorescent protein (RFP)-Fzr centriole marker, we examined centriole position in larval da neurons. We analyzed the ddaE neuron, which has a simple, stereotypic branching pattern [23]. As in developing motor neurons, centriole position varied in mature ddaE cells (Figure 1B). We quantified the position by dividing the neuron into four quadrants as done previously [20]. Using this method, quadrants 1 and 2 are always nearest to the axon, while quadrants 3 and 4 are always farthest from the axon. Centrioles were most frequently observed closer to the axon in quadrant 2 (around 50%, n = 10); however, the second most frequently populated quadrant was quadrant 4 (around 30%, n = 6), which is on the same side of the cell but farther from the axon (Figure 1B). Furthermore, the combined percentage total of centrioles closest to the axons (quadrants 1 and 2) was about 60% (n = 12) compared to 40% (n = 9) of centrioles positioned in quadrants farthest from the axon (quadrants 3 and 4). Thus, based on these percentages, there does not seem to be a strong positional bias of the centriole, as we did not observe consistent centriole localization in one part of the cell body in the embryo or larva.

### The core microtubule nucleation protein is not present at centrioles in embryonic or larval neurons

**Figure 2:**
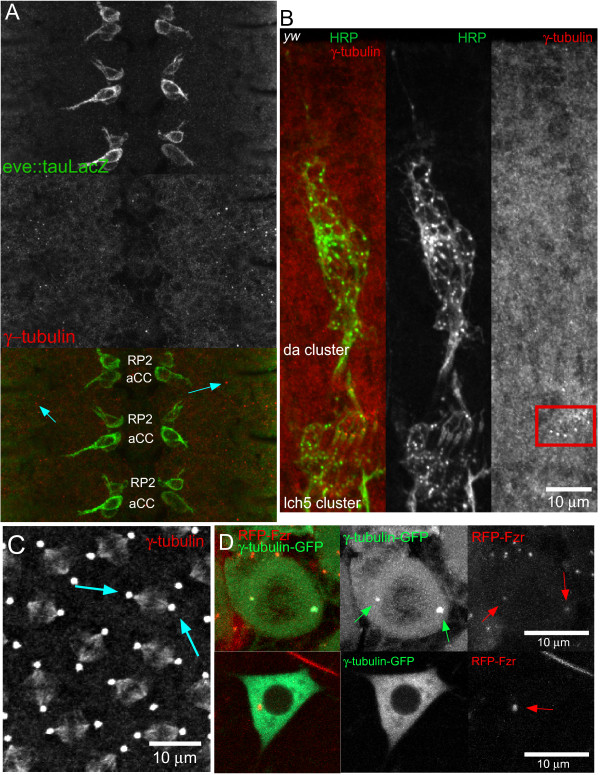
**The centriole does not accumulate γ-tubulin in developing and mature *Drosophila *neurons**. **(A) **Images of developing aCC and RP2 motor neurons expressing tau-LacZ and stained for γ-tubulin were taken in stage 12 embryos. Concentrated γ-tubulin was not seen in the neurons but was seen in distinct puncta in the surrounding epithelial cells (arrows), indicating the efficacy of the antibody. **(B) **Stage 16 embryos were stained with fluorescein isothiocyanate (FITC)-conjugated HRP and γ-tubulin antibody. Chordotonal neurons had γ-tubulin localization at the base of each cilium (red box), while no specific localization was seen in non-ciliated neurons. **(C) **Syncytial embryos were stained with γ-tubulin antibody. Dividing cells had γ-tubulin staining concentrated at the centrosome/spindle pole (arrows) and microtubules (between arrows) during metaphase. **(D) **Localization of both γ-tubulin-GFP and RFP-Fzr in third instar larvae is shown. Top: brains from larvae expressing γ-tubulin-GFP were examined after fixation. Concentrated areas of γ-tubulin-GFP localization (green arrows) were seen at spindle poles in dividing neuroblasts and show co-localization with RFP-Fzr (red arrows). Bottom: neurons in living larvae expressing γ-tubulin-GFP and RFP-Fzr were imaged. γ-Tubulin-GFP was diffuse throughout the neurons compared to RFP-Fzr, which was localized in a concentrated area (red arrow).

To test whether the centriole was likely to organize a functional centrosome and be a major site of neuronal microtubule nucleation, we examined γ-tubulin localization in both embryos and larvae. Since the centriole is known to be extremely important for organizing cilia, we used ciliated sensory neurons as positive controls in many of the experiments. These neurons have dendrites that consist of a single cilium [24], and thus polarized trafficking into these dendrites is controlled by intraflagellar transport. Moreover, the ciliated neurons and da neurons in which we analyzed microtubule polarity are born at similar times during embryogenesis. In stage 12 embryos, we performed immunostaining experiments using a γ-tubulin antibody. In motor neurons at the time of axon outgrowth, there were no distinct spots of γ-tubulin seen within the cells, even though γ-tubulin spots could be seen in surrounding cells (Figure 2A). To further examine γ-tubulin localization in neurons, we compared ciliated and non-ciliated neurons later in embryonic development. Neuronal membranes were stained with anti-horseradish peroxidase (anti-HRP) antibody [25], and the body wall of stage 16 embryos was imaged. Five ciliated neurons (lch5) make up the lateral chordotonal organ, which is very easy to identify due to its characteristic hand-like appearance. In these cells, γ-tubulin staining was seen concentrated at the base of the cilium, where the centriole or basal body sits, in each of the five neurons (n = 163 cells; red box, Figure 2B). In contrast, no spots of γ-tubulin localization were seen within the non-ciliated da neurons that lie dorsal to the chordotonal organ (n = 20; Figure 2B). To make sure we could reliably detect centrosomal γ-tubulin in stained embryos, we imaged mitotic cells in syncytial embryos; γ-tubulin was clearly visible at the spindle poles and on the spindle (Figure 2C).

In order to visualize γ-tubulin in living animals, we generated transgenic flies with a UAS-driven γ-tubulin-GFP. To determine whether this transgene localized like endogenous γ-tubulin, we examined it in dividing cells in conjunction with RFP-Fzr. Dividing neuroblasts can be observed in living larval brain explants [26] as well as fixed explants, and γ-tubulin-GFP was found concentrated at spindle pole centrosomes as expected in these cells (n = 10; green arrows, Figure 2D). These γ-tubulin-GFP concentrations co-localized with RFP-Fzr (red arrows, Figure 2D). However, in mature da neurons in living animals, no obvious puncta or concentrations of γ-tubulin-GFP could be identified (n = 7; Figure 2D). The only sensory neurons with obvious spots of γ-tubulin-GFP were those with cilia (not shown). The lack of γ-tubulin localization to neuronal centrioles in non-ciliated cells suggests they do not organize active centrosomes and are not sites of microtubule nucleation from very early in neuronal development.

### Trajectories of growing microtubules do not have a common source in da neurons

**Figure 3:**
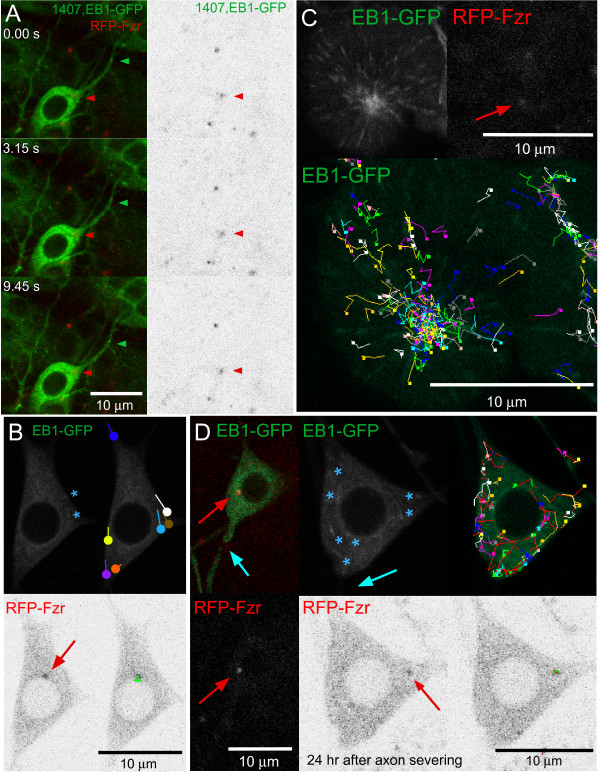
**Growing microtubules do not concentrate around the centriole in developing and mature *Drosophila *neurons, nor in neurons after axon severing**. **(A) **Microtubule orientation and dynamics during dendrite outgrowth were examined by tracking the movement of EB1-GFP in living embryos. EB1-GFP was expressed in neurons using 1407-Gal4. In the left images of a da neuron, an EB1-GFP comet can be seen to originate (green arrows) at an area away from the centriolar protein Fzr (right and left, red arrows), from where it traveled toward the cell body. The frames shown are from Additional file 1. Similarly, EB1-GFP movement in the cell body of da neurons can be seen to originate away from the centriole as well. This can be seen in Additional file 2. **(B) **Trajectories of growing microtubules in uninjured larval ddaE neurons show no origin at the centriole (arrow). EB1-GFP comets can be seen in the cell bodies of mature da neurons (top left, asterisks), although EB1-GFP dynamics are fairly quiet. EB1-GFP comets were tracked manually over multiple frames; MOSAIC Particle Tracker was used to track mRFP-Fzr. Trajectories were overlaid onto a single frame from Additional file 3. **(C) **Trajectories of growing microtubules were determined in larval neuroblasts. MOSAIC Particle Tracker for ImageJ was used to calculate EB1-GFP comet trajectories over multiple frames. In dividing neuroblasts, EB1-GFP comets produce a star-like pattern (top left), and their trajectories (bottom) originate at a common point from the centriole (arrow at top right). Trajectories were overlaid onto a single frame from Additional file 4. **(D) **Growing microtubules after axon severing of ddaE neurons do not associate with the centriole. Left: an example of an axon severed by a UV laser is shown. The blue arrow points to the site of injury, and the red arrow indicates the centriole. Twenty-four hours after severing, many EB1-GFP comets can be seen (asterisks). Tracking the comets and RFP-Fzr over multiple frames (right images) via MOSAIC Particle Tracker shows that the trajectories of the comets have no discernible origin point. Trajectories were overlaid onto a single frame from Additional file 5.

In cells with active centrosomes, microtubules can often be seen to emanate from their vicinity by plus-end tracking [27]. To test whether microtubule growth concentrates near the centriole, we performed live imaging of embryos expressing RFP-Fzr and GFP-tagged EB1, which binds to growing plus ends of microtubules. In embryonic da neurons during dendrite outgrowth, we observed new EB1-GFP comets emerging at various regions of the cell, including in dendrites far from the centrioles, which were predominantly localized to the cell body (n = 6 cells; Figure 3A; Additional file 1). In the cell body, EB1-GFP movement also emerged away from the centriole (n = 9 cells; Additional file 2). Mature larval neurons produce the same result; manually traced trajectories of EB1-GFP comets show no correlation with centriole position in the cell (n = 8 cells; Figure 3B; Additional file 3). This lack of specific pattern differs from dividing *Drosophila *cells, in which EB1-GFP can be seen to emerge in a star-shaped pattern from the centrosome (n = 19 cells; Figure 3C; Additional file 4) [28]. Indeed, by using MOSAIC Particle Tracker to determine the trajectories of these growing microtubules, we see that they come from a similar starting point at the centriole (Figure 3C). However, neuronal microtubules tend to be long, and so plus ends could initiate growth from stable microtubule shafts. To minimize this problem, we analyzed microtubule growth after axon injury when microtubules are predicted to be shorter.

Axon severing causes approximately a ten-fold increase in the number of microtubule plus ends in the cell body [29]. Since many more plus ends are present in the confined area of the cell body, each individual microtubule is predicted to be shorter, and thus the plus end should be closer to the site of nucleation. Again, using MOSAIC Particle Tracker, the trajectories of these growing microtubules were outlined, and growing microtubules were present throughout the cell (n = 17 cells; Figure 3D; Additional file 5). The increase in the number of growing microtubules could not be traced to one specific point, although there may be a possible preference for the cell and nuclear membranes (Figure 3D). Overall, these analyses indicate that centriole position does not correlate with sites of microtubule growth.

### Larval sensory neurons in *DSas-4 *mutants contain centrioles that are not fully functional

To test more rigorously whether centrioles are required for neuronal microtubule organization, we used *DSas-4 *mutations to disrupt the centriole. *DSas-4 *mutants lack centrioles in most cells due to loss of the centriole replication protein DSas-4 [22]. *DSas-4 *mutants begin embryogenesis with DSas-4 protein inherited from their mother. However, they do not make additional protein so that, during the course of embryonic cell divisions, the protein is depleted and centriole duplication ceases. At stage 15-16 of embryogenesis, more than half of the cells are reported to lack centrioles, and by late larval life, no brain cells had detectable centrioles [22].

**Figure 4:**
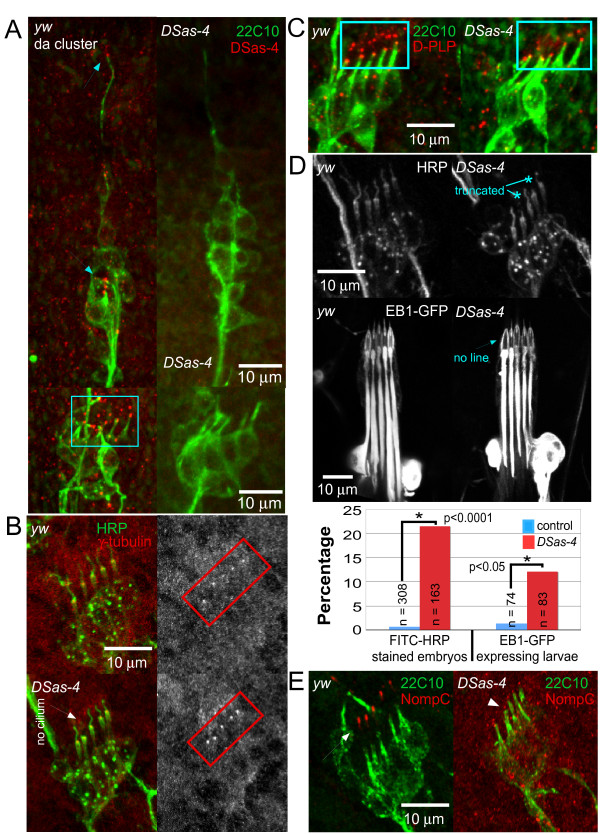
**Larval sensory neurons contain centrioles but have compromised centriole function**. *yw *(control) and *DSas-4 *embryos were fixed and stained with antibodies against either 22C10 to label stable microtubules or fluorescein isothiocyanate (FITC)-conjugated HRP to label neurons, β-galactosidase (to label heterozygote animals with a lacZ-marked balancer), and centriole-associated proteins. Stage 16 embryos were examined except where noted. **(A) ***yw *flies have punctate DSas-4 staining in ciliated neurons of the dorsal da cluster (arrows) and chordotonal neurons (box). *DSas-4 *embryos do not have any punctate DSas-4 staining. **(B) **Embryos were stained with FITC-conjugated HRP and γ-tubulin antibody. The γ-tubulin antibody localized at the base of each cilium in chordotonal neurons (boxes) of *DSas-4 *and *yw *animals. **(C) **Embryos were stained with antibodies against futsch (22C10) and the centriole-associated *Drosophila *pericentrin-like protein (D-PLP). D-PLP puncta were present in the chordotonal neurons of both control and *DSas-4 *mutants (boxes). Other centriolar proteins examined are seen in Additional file 6. **(D) **Cilia of chordotonal neurons were analyzed in *DSas-4 *and control animals. Top: embryos were stained with FITC-conjugated HRP. Middle: larvae expressing EB1-GFP in all neurons were examined by live imaging. Bottom: quantification of ciliary defects was performed. Neurons with abnormal cilia were classified as such by displaying either no cilium (arrow in (B), and arrow in the middle image of (D)) or a truncated cilium (asterisks, top image). *DSas-4 *mutants had a greater percentage of neurons with abnormal cilia compared to control samples. **(E) **Embryos were stained with antibodies against futsch and NompC. In control neurons, NompC localized to the distal tip of of mechanosensory cilia (arrow), while in *DSas-4 *mutants, NompC localization was seen at the base of the cilium (arrowhead).

As larval sensory neurons are most amenable to studies of microtubule organization, we wished to determine whether they lacked centrioles in *DSas-4 *mutants, as they have not previously been studied in these centriole duplication mutants. Larval sensory neurons, both ciliated and non-ciliated, are born mid-way through embryogenesis. As expected, in stage 16 embryos, *DSas-4 *mutant embryos lacked DSas-4 staining in sensory neurons, including ciliated chordotonal neurons (n = 75 cells; Figure 4A). However, when we stained using antibodies to other centriole-associated proteins, we found that many were still present. For example, γ-tubulin staining was consistently seen at the basal body in *DSas-4 *chordotonal neurons (n = 163 cells; Figure 4B). Furthermore, the *Drosophila *pericentrin-like protein (D-PLP) remained at the base of the cilium in *DSas-4 *mutants in both embryos (n = 75 cells; Figure 4C) and larvae (data not shown). Similar results were seen with Asterless (n = 75 cells) and Bld10 (n = 12 da clusters; Additional file 6). Thus, centrioles still seem to be present in most larval sensory neurons in *DSas-4 *mutants even though they did not contain detectable DSas-4, which is known to be essential for centriole assembly. To resolve this paradox, we further analyzed cilia structure in *DSas-4 *mutants, as centriole function is best understood and easiest to assess in these organelles.

Previous studies have found that loss of centriolar proteins leads to defects in cilia morphology [22,30]. To determine whether we could detect defects in chordotonal neurons despite the continuing presence of centrioles in many of the neurons in *DSas-4 *mutants, we analyzed cilia more closely in stage 16 embryos stained with fluorescein isothiocyanate (FITC)-HRP. We found that while most neurons had sensory cilia with normal morphology, about 20% of neurons had either no cilia (arrow, Figure 4B) or truncated cilia (asterisks, Figure 4D). We also examined larval chordotonal neurons expressing EB1-GFP. In almost all wild-type cilia, a thin line of EB1-GFP could be seen in the center of the ciliary dilation and extending out beyond its tip (Figure 4D). In *DSas-4 *mutant larvae, the number of cilia without this central line (arrow, Figure 4D) increased, indicating defects in assembly of the cilium (Figure 4D).

Since cilia morphology was defective in some larval sensory neurons in *DSas-4 *mutants, we wished to determine whether ciliary protein localization was disrupted in *DSas-4 *mutants. Previous studies have found that NompC, a transient receptor potential (TRP) ion channel, is localized at the distal tip of cilia in chordotonal neurons [31]. We stained control and *DSas-4 *embryos with 22C10 (anti-futsch, a neuronal microtubule-associated protein) and NompC antibodies. Control neurons displayed NompC localization at the distal tips as expected (n = 117 neurons; Figure 4E). Instead of localizing to the distal tip of the cilium in *DSas-4 *mutants, NompC staining was adjacent to 22C10 staining at the base of the cilium, indicating that ciliary transport was defective (n = 120 neurons; Figure 4E). Unlike the structural defects, which only manifested in a subset of ciliated neurons in *DSas-4 *mutants, the failure to transport NompC was observed in all ciliated neurons. We therefore conclude that even if the centriole is present in larval sensory neurons, it is not fully functional. This conclusion is consistent with a previous study that found that even though centrioles may be present when Sas-4 levels are partially reduced, they are defective and cannot perform all of their normal functions [32]. Although we performed this analysis in ciliated sensory neurons, all of the larval sensory neurons are born at a similar time during embryogenesis, and so were likely generated with similarly reduced levels of DSas-4 protein, and thus likely to have defective centrioles.

### Microtubule orientation remains unchanged in *DSas-4 *mutants

As larval sensory neurons lack DSas-4 protein and have defective centrioles in *DSas-4 *mutant animals, we used this genetic background to test whether a centriole-based centrosome might have any function in organizing the polarized arrays of microtubules in neurons. Dendritic arborization neurons have extremely polarized neuronal microtubules with close to 100% of axonal microtubules with plus ends distal to the cell body, and around 90 to 95% of dendritic microtubules with the opposite minus-end-out polarity, depending on the level of EB1-GFP expression [10,33]. To analyze microtubule polarity, we assayed the direction of EB1-GFP comet movement. Comets moving towards the cell body represent minus-end-out microtubules, and comets moving away from the cell body represent plus-end-out microtubules [8,10].

**Figure 5:**
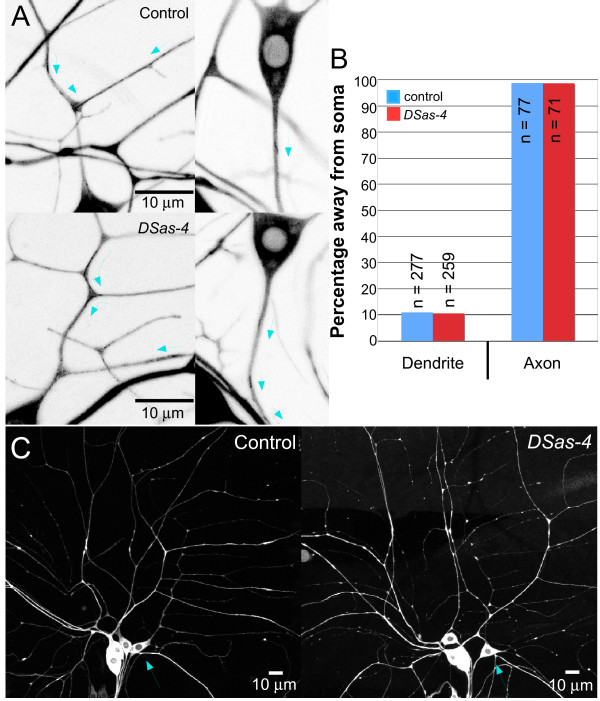
**Microtubule orientation in ddaE neurons of *DSas-4 *mutants is normal**. **(A) **EB1-GFP was expressed in neurons of *yw *and *DSas-4 *mutants using 109(2)80 Gal4. Movies of EB1-GFP movement were taken with a confocal microscope. For *yw *controls, a maximum projection of 41 frames into a single image was performed for the dendrite (top left), and a maximum projection of 7 frames was performed for the axon (top right). For *DSas-4 *mutants, a maximum projection of 59 frames into a single image was performed for the dendrite (bottom left), and a maximum projection of 18 frames was performed for the axon (bottom right). Arrows show the directionality of EB1-GFP movement. Additional files 7, 8, 9 and 10 show the data from which these figures originate. **(B) **The directionality of EB1-GFP comets was quantified in class 1 ddaE neurons. The 'n' values are the number of EB1-GFP comets analyzed. In all cases, *P *> 0.05 (Fisher's exact test), which indicates that the neurons retain their polarity despite loss of DSas-4. **(C) ***DSas-4 *mutants exhibit no changes in cell morphology. Live imaging was performed on control and *DSas-4 *larvae expressing heterozygous EB1-GFP,109(2)80. Dendritic and axonal structures remain similar in both cases. Blue arrows point to the cell body of the ddaE neuron.

In dendrites of control neurons, 89% of comets moved towards the soma (Figure 5A,B; Additional file 7). In axons, almost 100% of comets headed away from the soma (Figure 5A,B; Additional file 8). No significant difference in the direction of comet movement was found in *DSas-4 *mutants. With these mutants, in dendrites, 90% of comets moved towards the soma (Figure 5A,B; Additional file 9), while in axons, almost 100% of comets traveled away from the cell body (Figure 5A,B; Additional file 10). Additionally, morphology of the ddaE dendrites did not change (n = 7; Figure 5C). We conclude that neither a fully functional centrosome nor DSas-4 are required to maintain polarized arrays of neuronal microtubules.

### Elimination of the centriole using laser ablation does not affect microtubule orientation

Using *DSas-4 *mutants, we have shown that centrioles with disrupted function do not alter microtubule orientation. However, since the centriole persists in these early-born neurons, it may still play some type of role in da neurons.

**Figure 6:**
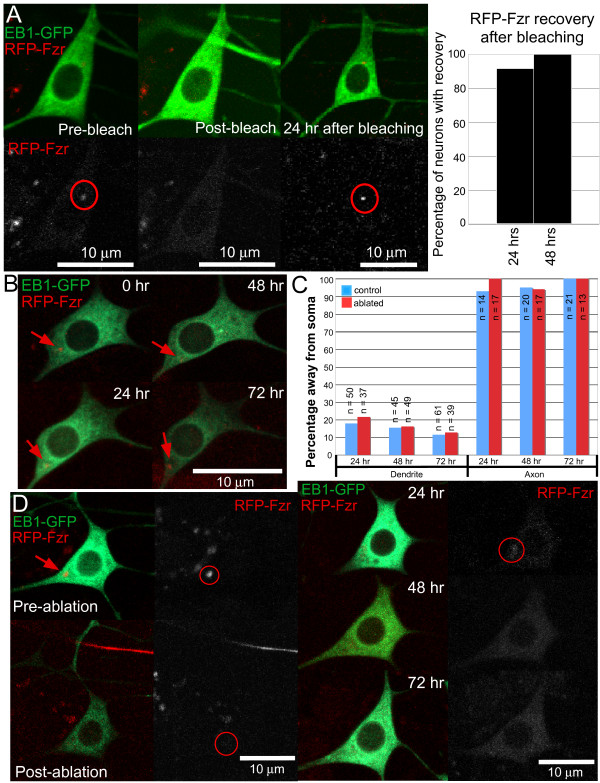
**Laser ablation of the centriole in larval ddaE neurons does not affect microtubule polarity**. **(A) **Bleaching of the centriole yields recovery of RFP-Fzr within 2 days. The red circle indicates the area containing the centriole that was bleached. Immediately post-bleach, no concentrated RFP-Fzr spot is seen compared to 24 hours post-bleach, at which time recovery has occurred (circle). As shown by the chart, recovery typically occurs within 24 hours (11 of 12 cells, and the remaining cell recovered by 48 hours). **(B) **Without laser ablation, the centriole persists in neurons. RFP-Fzr was imaged in ddaE neurons over a 72 hour time period. Six neurons from 6 larvae were imaged. Unlike centriole-ablated cells (Figure 6D), RFP-Fzr is clearly seen in the cell over all 3 imaging days. **(C) **Centriole ablation has no effect on the directionality of growing microtubules, which was quantified in class 1 ddaE neurons 1, 2, and 3 days after centriole ablation. The 'n' values are the number of EB1-GFP comets analyzed. Six neurons from 6 larvae were examined for both conditions. In all cases, *P *> 0.05 (Fisher's exact test), which indicates that the neurons retain their polarity despite ablation of the centriole. **(D) **Laser ablation eliminates RFP-Fzr concentrations. In this example, pre-ablation, the centriole is clearly seen in the cell body (circle). Post-ablation, the centriole appears to have fragmented. This is more clearly seen 24 hours post-ablation; past 24 hours, there are no noticeable concentrations of RFP-Fzr.

To address this issue, we eliminated the centriole from ddaE neurons expressing EB1-GFP and RFP-Fzr using a pulsed UV laser; this is the same technique used to sever axons (Figure 3D) [29]. One concern presented with this technique was that instead of successful ablation, bleaching of the centriole may actually occur. This was accounted for by bleaching RFP-Fzr concentrations to determine when RFP recovery would occur (Figure 6A). As seen in Figure 6A, in 11 out of 12 neurons, recovery occurred within a day, and the remaining cell showed recovery after 2 days. The results indicate that bleaching can be accounted for by recovery of RFP-Fzr signal within 2 days. Consequently, we decided to image once every day for 3 days past attempted ablation of the centriole, as no recovery of RFP signal by 72 hours would indicate successful ablation of the centriole.

In Figure 6B, without laser ablation of RFP-Fzr, the centriole persists through all imaging days (arrows). Generally, when laser ablation is performed, the RFP-Fzr concentration tends to fragment. The fragments are often seen 24 hours after ablation, and then the signal is completely diffuse after this point in time (Figure 6D). The directionality of EB1-GFP comets in both dendrites and axons were examined in 6 cells after centriole ablation. No significant changes in microtubule orientation were seen during any of the 3 days after ablation of the centriole (Figure 6C). In non-ablated control neurons, around 82% of EB1-GFP comets moved toward the soma at 24 hours. This percentage increased to around 85% after 48 hours, and by 72 hours, the percentage further increased to around 89%. Similarly, centriole-ablated neurons showed the same trend. Twenty-four hours after ablation of the centriole, around 80% of comets headed toward the soma. After 48 hours, an increase to 84% was seen, and by 72 hours, about 87% of comets moved in this direction. In the axon, microtubule orientation remained the same in both control and experimental neurons; in both cases, over 90% of comets headed away from the cell body during all three imaging days. These results confirm that the centriole is not required for the maintenance of microtubule organization in da neurons.

## Discussion

### The centrosome does not nucleate microtubules in developing or mature *Drosophila *neurons in vivo

The centrosome has been proposed to serve as the major source of neuronal microtubules [11]. However, we did not find any evidence that the centrosome could nucleate microtubules in *Drosophila *neurons. Very early in neuronal development, as axons emerge from the cell body, we did not find γ-tubulin concentrated at the centrosome (Figure 2A). Additionally, there were also no γ-tubulin puncta seen in the da neurons (Figure 2B), although they could be seen in mitotic cells (Figure 2C) and ciliated neurons (Figure 2B). We also did not find γ-tubulin-GFP concentrated in puncta in the cell body in mature neurons, nor was it co-localized with mRFP-Fzr (Figure 2D). These results are consistent with previous studies showing that nucleation sites are generally removed from the centriole as soon as mitosis is complete in *Drosophila *[28,34].

A similar course of events seems to occur in mammalian neurons, albeit on a delayed time frame. During axon specification in cultured hippocampal neurons, γ-tubulin localizes to the centrosome. At the time of dendrite outgrowth, less γ-tubulin is present on centrosomes, and less than half of the mature neurons have detectable γ-tubulin at centrosomes [16]. Earlier results, in which centrosomes were shown to be the major source of microtubule nucleation, were performed in immature neurons [12], and so are consistent with the conclusion that, in mature mammalian and *Drosophila *neurons, the centrosome is inactive and is not a major source of γ-tubulin-mediated microtubule nucleation.

### The centriole does not seem to be the source of growing microtubules

The centrosome has been shown to be important in organizing microtubules, specifically in forming polarized microtubule arrays such as the mitotic spindle [19]. However, in interphase cells, microtubule arrays can be formed independently of the centrosome [28], and even in proliferating cells, microtubule growth can be seen at acentrosomal poles and at mitotic spindles lacking proper centrosomes [35]. We find that microtubule growth in *Drosophila *da neurons did not concentrate near the centriole, even after axon injury (Figure 3A,B,D). There is an increase in the number of growing microtubules after injury; however, the paths of these growing microtubules did not correlate with centriole position (Figure 3D). This increase in growing microtubules seems to be due to nucleation (MC Stone, L Chen and MM Rolls, unpublished results); however, no increase in EB1-GFP comets near the centriole was seen. Thus, we conclude that the centriole does not seem to have a major role in initiating microtubule growth.

### The centrosome does not play a role in maintaining neuronal microtubule organization

The centrosome has been proposed to serve as the major source of new neuronal microtubules, which could then be transported in a polarized manner to axons and dendrites [11,15]. This is similar to a model of microtubule organization in skin cells, in which microtubule minus ends are nucleated at the centrosome and then removed to the plasma membrane, where they are anchored by ninein [36]. Although we have found that the centrosome does not seem to be a major nucleator in *Drosophila *neurons, it could serve a role in maintaining the arrangement of microtubules in the cell. In this case, the centriole may be required to have a fixed location in the cell. We examined centriole position in developing motor neurons, and found that the centriole exhibited variable positioning in the cells (Figure 1A). Centriole positioning was variable even at the time of axon outgrowth, at which time the centriole localizes to the base of the nascent axon in some cells [20]. However, this corresponds with *in vivo *results in retinal ganglion cells and rhombic lip-derived neurons in zebrafish, where the centrosome was not adjacent to the site of the emerging axon [37,38]. The centriole also did not occupy a stereotyped location in mature da neurons (Figure 1B).

We examined microtubule orientation in *DSas-4 *mutants, which have been characterized as having a gradual loss of centrioles until full depletion in late larval life [22]. In flies, centrioles have been shown to be essential in very early embryonic development, but dispensable for later development [22,39]. In studies such as these, the tested cell types tended to be either later-born cells, which would have no maternal protein contribution, or cells that have undergone multiple divisions, in which the protein will be lost. Our larval sensory neurons are early-born and do not undergo further cell division, so there was a possibility that centrioles were capable of being formed early on. Our studies show that the centriole is still present in many larval sensory neurons, although DSas-4 protein is undetectable (Figure 4A-C; Additional file 6). *DSas-4 *mutants have also been characterized as completely lacking cilia or flagella. We find that the structure of the cilium is normal in at least three-fourths of the chordotonal neurons (Figure 4D). However, NompC, a mechanosensory transduction channel, is unable to be trafficked into the cilia of chordotonal neurons in *DSas-4 *embryos (Figure 4E). This is likely due to alterations in microtubule structure in the cilium, as intact microtubules are required for ankryin repeat-mediated NompC association and localization [31]. Thus, although centrioles remain in early-born sensory neurons, they do not retain full function. This is consistent with the finding that DSas-4 is required for pericentriolar material recruitment, as it is needed to scaffold and tether pericentriolar material components to the centrosome, and that lack of DSas-4 does not result in a complete lack of centriolar structures [40]. Instead, lack of DSas-4 can produce unstable procentrioles and centrosomes that are not fully functional [40]. Even though the centriole is likely to be partially disrupted in all embryo-born sensory neurons, we did not observe defects in neuronal organization or cell morphology in non-ciliated neurons, even at the level of microtubule orientation, which has not previously been examined in neurons of *DSas-4 *mutant animals (Figure 5).

Although the centriole does not seem to be fully functional in embryo-born sensory neurons, there remained the possibility that it could still contribute to microtubule organization in some way. In previous studies, centriole function has been probed by using laser ablation [16,41]. We therefore decided to use this approach to eliminate the centriole. No defects in microtubule organization in either dendrites or axons were found, even when imaging 3 days past ablation (Figure 6C). We conclude that the centrosome is not required for normal neuronal microtubule organization.

## Conclusion

In this paper, we have studied the role of the centrosome in maintaining microtubule organization in *Drosophila *neurons. We have found that the centriole does not organize a functional centrosome in developing or mature neurons, nor does it contribute to the increase in growing microtubules after axon injury, and that the centrosome is not required to maintain microtubule polarity in larval sensory neurons. Several important questions emerge from these conclusions, including: what nucleates microtubules in neurons, and where are nucleation sites localized? How is overall microtubule polarity established and maintained in neurons?

We propose that after mitotic exit, nucleation sites are relocalized to dispersed sites in neurons, as in other *Drosophila *cells [28,34]. These proposed nucleation sites are likely smaller, especially if they are localized in dendrite branches, which are very thin, and are likely much less active in mature neurons, where EB1 dynamics are quieter compared to dividing cells. Consequently, these nucleation sites are likely to contain less γ-tubulin than centrosomal nucleation sites, and thus may not be detectable by light microscopy. Where these nucleation sites are localized could be very important for determining microtubule polarity, particularly in dendrites in which microtubule minus ends are oriented away from the cell body. One potential site of microtubule nucleation is the Golgi complex. It has been shown to nucleate microtubules in mammalian cells [42], and possibly also in cultured *Drosophila *cells [28]. Additionally, although the centrosome as a whole does not play a role in microtubule polarity, we do not rule out that centrosome-associated proteins may be involved in microtubule dynamics and orientation. Depletion of the Mini spindles protein has been shown to eliminate EB1-GFP dynamics in *Drosophila *neurons [29], and γ-tubulin contributes to acentrosomal microtubule nucleation in other cell types [16,28]. Determining whether centrosomal proteins, the Golgi, or another organelle houses nucleation sites in dendrites, or whether microtubules are nucleated at dispersed sites in the cell body and then transported to axons or dendrites, will be critical for understanding how microtubule organization is established in neurons.

## Materials and methods

### *Drosophila *stocks

The Bloomington *Drosophila *Stock Center provided the following stocks: elav-Gal4 driver flies, 109(2)80 driver flies, 221-Gal4 driver flies, and 1407 driver flies. *DSas-4^S2214^*/TM6C and *DSas-4^S2214^*/TM3_ftzlacz _flies were obtained from Jordan Raff (University of Oxford). Miki Fujioka and Jim Jaynes (Thomas Jefferson University) provided the *eve::tau-lacZ *line, and Renata Basto and J Raff provided the GFP-Fzr and mRFP-Fzr lines. WeeP^304^tau-GFP and UAS-EB1-GFP lines were described previously [10,43].

We generated the UAS-γ-tubulin-GFP transgenic fly line as follows. UAS-γ-tubulin GFP was generated by amplifying the γ-tubulin 23C coding sequence from the *Drosophila *Genomics Resource Center cDNA clone LD40196 using forward primer 5'-ACTTGCCTCGAGCAAAACATGCCAAGTGAAATAATTACTTTGCAG-3' to introduce an XhoI site at the 5' end and reverse primer 5'-CATCGAGCTAGCTCCCGTGGAACCGGCGCTGGTCACAGATCG-3' to introduce an Nhe I site at the 3' end. The γ-tubulin 23C coding sequence was then inserted into the polylinker of a modified pUAST vector that includes a single copy of emerald GFP after the polylinker. UAS-γ-tubulin GFP plasmid was injected into embryos by the Massachusetts General Hospital *Drosophila *Core, and transgenic flies were generated using standard procedures.

### Axon severing and centriole ablation

To image microtubule growth after axonal injury, 221-Gal4, UAS-EB1-GFP and mRFP-Fzr lines were crossed to each other. For *in vivo *localization experiments in da neurons, embryos were collected at room temperature for 24 hours on apple juice agar caps. They were then transferred to food vials and incubated at 25°C for 48 to 72 hours. Larvae were mounted on 3% agarose dried to microscope slides and the coverslip taped down to prevent excessive movement. Axon severing of the class I ddaE neuron was performed using a pulsed UV laser (Photonic Instruments, Saint Charles, IL, USA). Imaging of the neuron was performed immediately after ablation to confirm that the axon was successfully cut. Larvae were then placed back into food and imaged the following day. Imaging was performed on a Zeiss LSM 510 confocal microscope, and all images were analyzed using ImageJ. EB1-GFP comet trajectories were determined manually (Figure 3B) or using the MOSAIC Particle Tracker plug-in for ImageJ (all other trajectories). The detection and tracking algorithms used for the MOSAIC plug-in are described in [44].

Similarly, the UV laser was also used for centriole ablation experiments. Larvae were imaged before ablation, immediately following ablation, and then placed back into food. They were then imaged 24, 48, and 72 hours after, with placement back into food between each imaging session. Imaging was performed on a Zeiss LSM 510 confocal microscope. For EB1-GFP directionality assays, only the comb-like dendrite 1 (labeled according to [29]) of the ddaE neuron was analyzed. For both control and centriole ablation experiments, 6 neurons from 6 larvae were imaged and analyzed; 'n' values indicate the number of EB1-GFP comets able to be tracked over 3 consecutive frames. Directionality of EB1-GFP comets was manually determined by movement away from or toward the soma in the dorsal dendritic trunk and axon. Control experiments to determine whether the centriole was simply bleached instead of ablated were performed on an Olympus FluoView™ FV1000 confocal microscope with bleaching performed with a 405 nm laser. Bleaching of the centriole area occurred over 3 to 5 seconds. Images were acquired before and immediately after bleaching of mRFP-Fzr in neurons. Larvae were placed back into food and examined every day after to determine when recovery occurred.

### Immunostaining procedures

For immunostaining of embryonic motor neurons, embryos from GFP-Fzr flies crossed to *eve::tau-lacZ *flies were used. For studies in ciliated neurons, the *DSas-4^S2214^*/TM3_ftzlacz _line was used, with *yw *flies as a control. Embryos were collected at room temperature for 24 hours on apple juice caps. Embryos were dechorionated for 2 minutes with 50% bleach and washed with distilled water for 3 minutes. They were fixed for 20 minutes with 450 μl PBS/50 μl formaldehyde (36.5%; Sigma, St Louis, MO, USA)/500 μl heptane. After fixation, the lower phase was removed, and 500 μl methanol was added to the tube, with vigorous shaking for 50 seconds. Embryos were washed with 1.5 ml methanol for 10 minutes, then with 1.5 ml 0.1% Triton X-100 in PBS, and then twice with 1.5 ml blocking solution (PBS/1% bovine serum albumen/0.2% Triton X-100/10 mM glycine). Embryos were incubated overnight at 4°C with primary antibodies in blocking solution. They were then washed in blocking solution for several hours before being incubated for 2 hours with secondary antibodies, washed for at least an hour, and equilibrated overnight in 85% glycerol/50 mM Tris, pH 8. Homozygous *DSas4 *mutants were identified based on lack of β-galactosidase staining.

Primary antibodies used were: mouse anti-γ-tubulin (Sigma), rabbit anti-γ-tubulin (Sigma), 22C10 (Developmental Studies Hybridoma Bank), FITC-conjugated rabbit anti-HRP (Jackson Immunoresearch, West Grove, PA, USA), chicken anti-β-galactosidase (Gallus Immunotech, Fergus, ON, Canada), rabbit anti-DSas-4 (gift from J Raff), rabbit anti-Asterless (gift from J Raff), rabbit anti-Bld10 (gift from T Megraw), rabbit anti-D-PLP (gift from Greg Rogers), and rabbit anti-NompC (gift from Li Cheng and Yuh Nung Jan). Secondary fluorescent antibodies were obtained from Jackson Immunoresearch. Imaging was performed on an Olympus FluoView™ FV1000 confocal microscope.

### Fixation and live imaging of larval brains

For γ-tubulin-GFP localization studies in dividing neuroblasts, elavGal4/CyO flies were crossed to UAS-γ-tubulin-GFP flies, and third instar larval brains from GFP-expressing larvae were dissected in Schneider's medium and fixed with 4% paraformaldehyde. The brains were then washed in blocking solution (see above) for at least one hour and equilibrated in 85% glycerol/50 mM Tris, pH 8. For live imaging of EB1-GFP dynamics in larval neuroblasts, elavGal4, UAS-EB1-GFP flies were crossed to RFP-Fzr flies. After larval brains were dissected, they were placed with a drop of PBS onto a gas-permeable membrane (Yellow Springs Instruments, Yellow Springs, OH, USA). The membrane was placed on one side of a chamber slide, and then a coverslip was placed over it. For imaging of larval brains, at least 5 brains were dissected and analyzed. Imaging was performed on a Zeiss LSM 510 confocal microscope.

### Live imaging of embryos

1407,EB1-GFP;RFP-Fzr flies were crossed to RFP-Fzr flies to maintain low EB1-GFP expression while having maximal RFP-Fzr expression. Embryos were collected and dechorionated as described above. They were then transferred to a tube containing 500 μl heptane for easier management. Embryos were spread out over a coverslip and covered in halocarbon oil 27 (Sigma). The coverslip was then attached to a metal slide with an opening to view them. Imaging was performed on a Zeiss LSM 510 confocal microscope.

### Localization and quantification experiments

For *in vivo *localization experiments in da neurons, embryos were collected at room temperature for 24 hours on apple juice agar caps. They were then transferred to food vials and incubated at 25°C for 72 hours. As a result, third instar larvae were imaged 72 to 96 hours after egg laying. Larvae were mounted on 3% agarose dried to microscope slides and the coverslip taped down to prevent excessive movement. To look at centriole localization, RFP-Fzr/CyO;WeeP^304^tau-GFP flies were used. Imaging was performed on a Zeiss LSM 510 confocal microscope. Centriole localization was quantified using a similar method to [20]. The centroid of the nucleus of the neuron was determined, and then axes were drawn to separate the soma into four quadrants, with one axis parallel to the initial region where the axon first extends (Figure 1B).

For γ-tubulin studies in mature neurons, elavGal4,γ-tubulin-GFP/CyO flies were crossed to RFP-Fzr flies, and images were acquired on the Olympus FluoView™ FV1000 confocal microscope. For cilia morphology studies in chordotonal neurons, elavGal4/CyO;*DSas-4*/TM6 flies were crossed to UAS-EB1-GFP;*DSas-4*/TM6 flies, and GFP-expressing non-tubby larvae were picked and imaged. To serve as a control, elavGal4/CyO flies were crossed to UAS-EB1-GFP flies, and GFP-expressing larvae were picked and imaged. For analysis and quantification of cilia morphology of the chordotonal neurons, neurons were grouped into one of two categories: with cilia or with no/abnormal cilia. At least 8 larvae were imaged for each cross. This analysis and quantification was also used for neurons of *yw *and *DSas-4 *embryos stained with FITC-HRP, in which at least 10 embryos were imaged for each line.

For experiments on microtubule orientation in da neurons, EB1-GFP,109(2)80;*DSas-4*/TM6 flies were crossed to *DSas-4*/TM6C flies, and non-tubby larvae were picked and imaged. EB1-GFP,109(2)80 flies were crossed to *yw *flies as a control. EB1-GFP comets were assayed using the same method as the centriole ablation experiments. At least 10 larvae were imaged for each cross; 'n' values indicate the number of EB1-GFP comets able to be tracked over 3 consecutive frames. To image morphology of the da cluster, a Z-projection was performed. Imaging was performed on a Zeiss LSM 510 confocal microscope. Images were analyzed using ImageJ.

## Abbreviations

da: dendritic arborization; D-PLP: *Drosophila *pericentrin-like protein; FITC: fluorescein isothiocyanate; GFP: green fluorescent protein; HRP: horseradish peroxidase; PBS: phosphate-buffered saline; RFP: red fluorescent protein.

## Competing interests

The authors declare that they have no competing interests.

## Authors' contributions

MMN performed all immunostaining, localization, and EB1-GFP experiments, except specified below. MCS generated UAS-γ-tubulin-GFP flies. MMR performed fixation and immunostaining experiments on embryonic motor neurons and supervised in the planning of experiments and the writing process.

## Author information

MMR is a Pew Scholar in the Biomedical Sciences.

## Supplementary Material

Additional file 1 **Movie 1**. EB1-GFP dynamics in dendrites of da neurons of embryos expressing RFP-Fzr. Click here for file

Additional file 2 **Movie 2**. EB1-GFP dynamics in the cell body of da neurons of embryos expressing RFP-Fzr. Click here for file

Additional file 3 **Movie 3**. EB1-GFP dynamics in an uninjured larval ddaE neuron expressing RFP-Fzr. Click here for file

Additional file 4 **Movie 4**. EB1-GFP dynamics in larval neuroblasts. Click here for file

Additional file 5 **Movie 5**. EB1-GFP dynamics in a ddaE neuron expressing RFP-Fzr 24 hours after axon severing. Click here for file

Additional file 6 **Figure S1**. Late-stage embryos (stage 16) were stained with antibodies against 22C10 and the centriole-associated proteins Asterless (Asl) and Bld10. The localization of the centriolar proteins (boxes and arrows) do not differ in control and *DSas-4 *mutants. Click here for file

Additional file 7 **Movie 6**. EB1-GFP dynamics in the dendrite of a class 1 ddaE neuron (control). EB1-GFP was expressed with one copy of 109(2)80. Click here for file

Additional file 8 **Movie 7**. EB1-GFP dynamics in the axon of a class 1 ddaE neuron (control). EB1-GFP was expressed with one copy of 109(2)80. Click here for file

Additional file 9 **Movie 8**. EB1-GFP dynamics in the dendrite of a class 1 ddaE neuron from a *DSas-4 *mutant. EB1-GFP was expressed with one copy of 109(2)80. Click here for file

Additional file 10 **Movie 9**. EB1-GFP dynamics in the axon of a class 1 ddaE neuron from a *DSas-4 *mutant. EB1-GFP was expressed with one copy of 109(2)80. Click here for file
