# Supplementary figures and images for: Microtubules are organized independently of the centrosome in Drosophila neurons
Source: Neural Dev. 2011 Dec 6;6:38. doi: 10.1186/1749-8104-6-38 (PMC3271965; doi:10.1186/1749-8104-6-38)

A

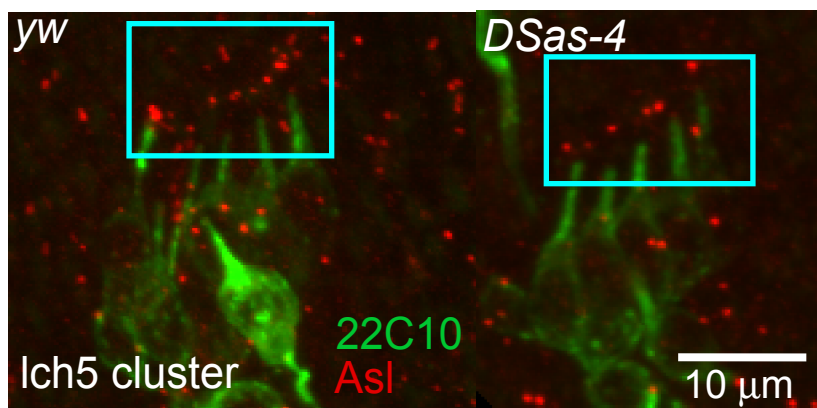

B

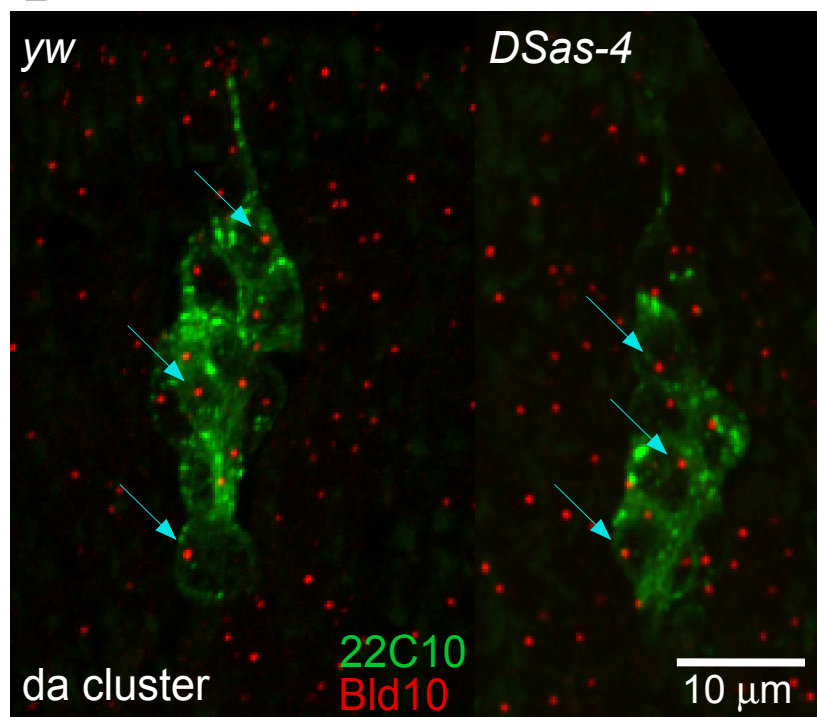

Supplement: Additional file 6 — Figure S1. Late-stage embryos (stage 16) were stained with antibodies against 22C10 and the centriole-associated proteins Asterless (Asl) and Bld10. The localization of the centriolar proteins (boxes and arrows) do not differ in control and DSas-4 mutants. [file 1749-8104-6-38-S6.PDF]
